# Advances in Prenatal Diagnosis of Placenta Accreta Spectrum

**DOI:** 10.3390/medicina61030392

**Published:** 2025-02-24

**Authors:** Qiuming Chen, Kuifang Shen, Yating Wu, Jianling Wei, Jingrui Huang, Chenlin Pei

**Affiliations:** 1 Department of Obstetrics, Xiangya Hospital Central South University, 87 Xiangya Road, Changsha 410008, China; 2 Hunan Engineering Research Center of Early Life Development and Disease Prevention, Changsha 410008, China

**Keywords:** prenatal diagnosis, placenta accreta spectrum, ultrasound

## Abstract

Placenta accreta spectrum (PAS) involves abnormal placental attachment and can lead to severe complications such as postpartum hemorrhage and hysterectomy. Ultrasound is the main tool used to screen for PAS due to its non-invasive nature and convenience, although its accuracy depends on the skill of the operator. Magnetic Resonance Imaging has emerged as a supplementary tool, especially for complex cases or posterior placentas, providing more accurate anatomical detail and enabling the invasion depth and location to be assessed. This review summarizes recent advances in prenatal imaging for PAS, aiming to improve diagnostic accuracy and guide future research.

## 1. Introduction

Placenta accreta spectrum (PAS) refers to abnormal placental adherence or invasive growth into the uterine myometrium. It is classified into three grades based on the degree of invasion: placenta accreta, placenta increta, and placenta percreta [1]. According to recent studies, the average incidence of PAS is approximately 0.17%, equating to 1–2 cases per 1000 deliveries [2]. However, in high-risk populations, such as those with multiple cesarean sections, those assisted by reproductive technologies, and those with obesity, the incidence is notably higher [3,4]. Approximately 50% of PAS cases remain undiagnosed prior to delivery [5], significantly exacerbating the risks of adverse maternal and fetal outcomes. For mothers, the disorder increases the risk of postpartum hemorrhage, organ dysfunction, and hysterectomy. The severity of PAS directly correlates with increased bleeding during surgery, leading to a greater need for blood transfusions. For neonates, PAS is associated with an increased risk of preterm birth, intrauterine growth restriction (IUGR), and respiratory distress syndrome (RDS) due to placental insufficiency. Additionally, PAS is associated with a higher rate of neonatal intensive care unit (NICU) admissions and long-term developmental delays [4]. These adverse maternal and neonatal outcomes highlight the importance of the early and accurate diagnosis of PAS, which is crucial for guiding effective multidisciplinary management. A well-coordinated approach that involves obstetricians, maternal–fetal medicine specialists, anesthesiologists, radiologists, and blood bank specialists can prevent the occurrence of severe complications. Early diagnosis enables timely intervention and helps ensure that the appropriate professionals are involved in the management of this condition, ultimately improving outcomes for both mothers and neonates [6].

Improving maternal and fetal outcomes depends upon the early and accurate diagnosis of PAS. Ultrasound, including 2D, color Doppler, and 3D imaging, remains the primary screening tool employed due to its non-invasive nature, widespread availability, and convenience. However, ultrasound’s diagnostic accuracy is highly dependent on the operator’s experience and is limited by the quality of the equipment used. Furthermore, there is a lack of standardized protocols, which could lead to variability in diagnostic outcomes. Recently, Magnetic Resonance Imaging (MRI) has emerged as a valuable supplementary tool, especially in complex cases or when the placenta is located posteriorly; in these situations, the use of ultrasound may be limited [7]. MRI provides enhanced anatomical detail and is particularly useful for assessing the depth of invasion and the relationship between the placenta and surrounding organs. Despite its advantages, MRI also has its limitations, such as its high cost, longer scanning time, and reliance on specialized equipment and expertise.

This review summarizes recent advances in prenatal imaging for PAS, with a focus on improving diagnostic accuracy and highlighting the complementary roles of ultrasound and MRI. By enhancing our understanding of these imaging techniques and their limitations, we can improve clinical management, minimize complications, and ultimately improve both maternal and neonatal outcomes.

## 2. Pathophysiology and High-Risk Factors for Placenta Accreta Spectrum

### 2.1. The Pathogenesis of Placenta Accreta Spectrum (PAS)

The pathogenesis of placenta accreta spectrum (PAS) involves a variety of physiological and pathological factors that may act independently or in concert to contribute to its development. Due to this complexity, the mechanisms underlying PAS are not yet fully understood. Core processes include decidual defects, abnormal placental attachment, and dysregulated angiogenesis and growth factors [8,9]. The factors that increase the risk of PAS include the following: 1. uterine scars and a history of cesarean sections; 2. placenta previa; 3. an advanced maternal age; 4. multiparity (multiple pregnancies); 5. the use of assisted reproductive technologies; and 6. endometrial damage. These factors can compromise the decidua, leading to abnormal placental invasion into the myometrium [10]. Understanding these mechanisms is essential for improving diagnostic approaches and clinical management strategies.

### 2.2. Prenatal Imaging Diagnosis of Placenta Accreta Spectrum

The pathological diagnosis of PAS depends on the microscopic examination of placental villi and their attachment or invasion into the uterine myometrium. Since placental pathology sampling is typically limited to specimens from uterine excision or partial resection, a full evaluation of placental implantation can only be performed after surgical removal [1].

**Figure 1:**
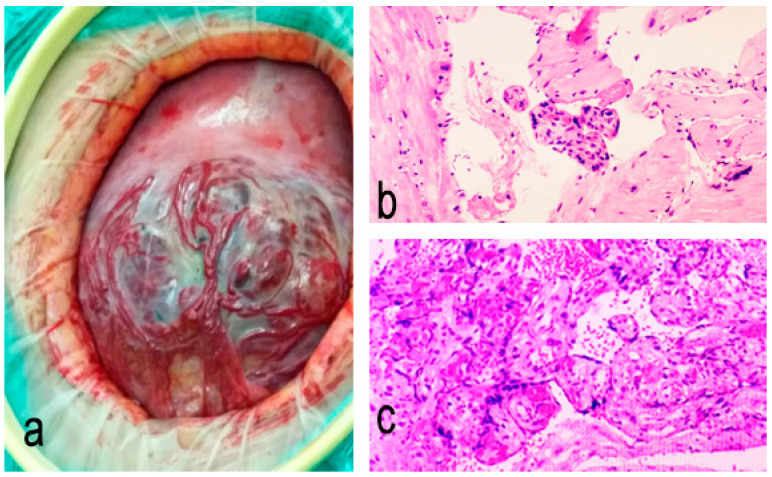
Intraoperative and pathological images of PAS: (**a**) intraoperative image; (**b**,**c**) histological images.

Histopathological examination can yield different results in the same patient depending on the relevant tissue, especially when focal PAS is present during conservative surgery. In severe PAS cases, particularly placenta percreta, the histopathologist may face difficulties in interpreting the specimen due to the remaining myometrium being too thin. This highlights the limitations of histopathology in diagnosing PAS when there is minimal viable tissue left for examination. Therefore, early imaging with ultrasound and MRI becomes crucial, as it enables the detection of placental invasion before tissue is removed, thus guiding surgical planning and minimizing the reliance on post-surgical histopathology for critical diagnoses (Figure 1).

## 3. Ultrasound in Placenta Accreta Spectrum

Ultrasound is the preferred tool for the prenatal diagnosis of PAS and plays a significant role throughout various stages of pregnancy. The integration of 2D grayscale ultrasound, color Doppler, and 3D ultrasound enables an accurate assessment of the type and extent of placental invasion. These modalities provide critical information that supports individualized planning and management, enabling preparations to be made against potential complications and improving maternal and neonatal outcomes [5].

### 3.1. Early Pregnancy (<14 Weeks): Early Screening and Risk Assessment

#### 3.1.1. Specific Ultrasound Signs

Recent studies have demonstrated that PAS may exhibit specific ultrasound signs during the first trimester of pregnancy [5]. Below are the key ultrasound features observed at different stages of early pregnancy:

At 6–8 weeks, ultrasound is particularly valuable in identifying PAS, especially in cases of caesarean scar pregnancies (CSPs). CSP is often a precursor to PAS, as the placental attachment within the cesarean scar can evolve into a deeper invasion if not monitored closely. CSP typically presents at 6–8 weeks, as the gestational sac is located at or near the cesarean scar; this is a key ultrasound feature used for early diagnosis [11,12]. The features observed at this stage include the following: 1. Gestational Sac Location: Low implantation near a cesarean scar is a common early sign of PAS [2]. 2. Placental Lacunae: Anechoic or hypoechoic areas are indicative of possible invasive attachment [13]. 3. Myometrial Thinning: A reduction in myometrial thickness (typically less than 5 mm) is a strong indicator of PAS [14].

At 11–13 weeks, ultrasound continues to serve as the primary diagnostic tool, but detecting PAS becomes more challenging due to the development of the placenta. The specific features used to observe PAS include the following: 1. Gestational sac location: Low implantation may still be present, but it is less distinct than that in the earlier stages of pregnancy [2,12]. 2. Placental abnormalities: Subtle signs of placental invasion may be detected, but these signs are often less distinct than those at earlier stages [12]. 3. Increased vascularity: Using color Doppler, increased vascularity in the placental area may suggest abnormal trophoblastic invasion; however, this is harder to assess at 11–13 weeks [2,12].

High-resolution transvaginal ultrasound (TVS) is generally used during early pregnancy due to its ability to provide a clear visualization of the gestational sac and myometrial thickness, particularly in cases of low-lying gestational sacs and during the early detection of local invasion [14]. Color Doppler ultrasound is utilized to detect abnormal blood flow signals within the placental area; abundant blood flow can suggest the presence of an invasive placenta, aiding in risk assessment.

#### 3.1.2. Challenges

Although ultrasound is frequently used for the early screening of PAS, it has limitations that may affect its diagnostic accuracy and consistency: 1. Low sensitivity: While a low-lying gestational sac serves as an important early warning sign, the ability of ultrasound to detect this during early pregnancy is limited, meaning that some cases of PAS may be missed during initial ultrasound screenings [15]. 2. Low accuracy: Placental lacunae are present in only 46% of cases during early pregnancy. Given the dynamic changes in the structure of the placenta and the gestational sac location throughout early gestation, this variability can lead to inconsistent findings, reducing the diagnostic accuracy [2]. 3. Specificity and false positives: The specificity of certain ultrasound signs is limited and can result in false positives. For instance, the detection of non-specific blood flow signals by color Doppler can occur in both abnormal and normal pregnancies, particularly in cases with placenta previa or threatened miscarriage [15]. 4. Operator experience and equipment quality: The accuracy of ultrasound in diagnosing PAS depends on the operator’s experience and the quality of the equipment. Inexperienced operators may miss subtle signs such as myometrial thinning or placental lacunae. Additionally, low-quality or outdated ultrasound machines may provide unclear images, leading to missed diagnoses or misinterpretations. Therefore, skilled operators and high-quality equipment are required to achieve accurate diagnosis [12].

### 3.2. Second and Third Trimester

Ultrasound examination during the second and third trimesters is crucial in screening for PAS. By this stage, features indicative of invasive implantation begin to manifest more clearly. This period is essential for identifying and evaluating the risk of PAS, as the detailed visualization of placental location and morphology can provide valuable diagnostic information [5].

#### 3.2.1. Common Ultrasound Signs (2D)

The disappearance of the hypoechoic zone behind the placenta: In a healthy pregnancy, there is typically a distinct hypoechoic area behind the placenta. The partial or complete absence of this zone suggests the possibility of PAS, indicating abnormal placental invasion into the myometrium. Placental lacunae: Multiple irregular anechoic or hypoechoic areas within the placenta, which are often referred to as placental lacunae or the “Swiss cheese sign”, are important markers of placental invasion. Placental Bulge: A placental protrusion into the bladder or other adjacent organs may indicate placenta percreta [3,13,15].

#### 3.2.2. Common Ultrasound Signs (Color Doppler)

Abnormal blood flow signals: Enhanced blood flow and the presence of bridging vessels at the uterine–placental interface may suggest invasive placentation [16]. Bladder wall blood flow abnormalities: High blood flow signals at the uterine–bladder interface indicate that the placenta may have penetrated the uterine wall and invaded the bladder. Bridging vessels at the uterine–bladder junction: The presence of parallel vessels at the uterine–bladder interface, connected by vertical vessels, suggests deep invasive placentation [13,17,18].

#### 3.2.3. Common Ultrasound Signs (3D)

Three-dimensional ultrasound provides a detailed representation of the placental structure and its relationship with the uterine wall, which is particularly useful for assessing complex cases such as posterior placental invasion. Second-trimester ultrasound can assess the depth and extent of placental invasion, offering crucial information for delivery planning and further screening. Compared to earlier stages, mid-pregnancy ultrasound is more sensitive in detecting invasive features such as myometrial thinning and placental bulge [10]. It also allows for the observation of abnormal blood flow using color Doppler, which is pivotal in determining whether multidisciplinary management is required.

### 3.3. Ultrasound Scoring Systems for Placenta Accreta Spectrum

Traditional ultrasound has limitations in detecting invasive placentation. To improve its diagnostic accuracy, researchers have developed several scoring systems that quantify multiple ultrasound features to systematically evaluate the risk of PAS [19].

**Figure 2:**
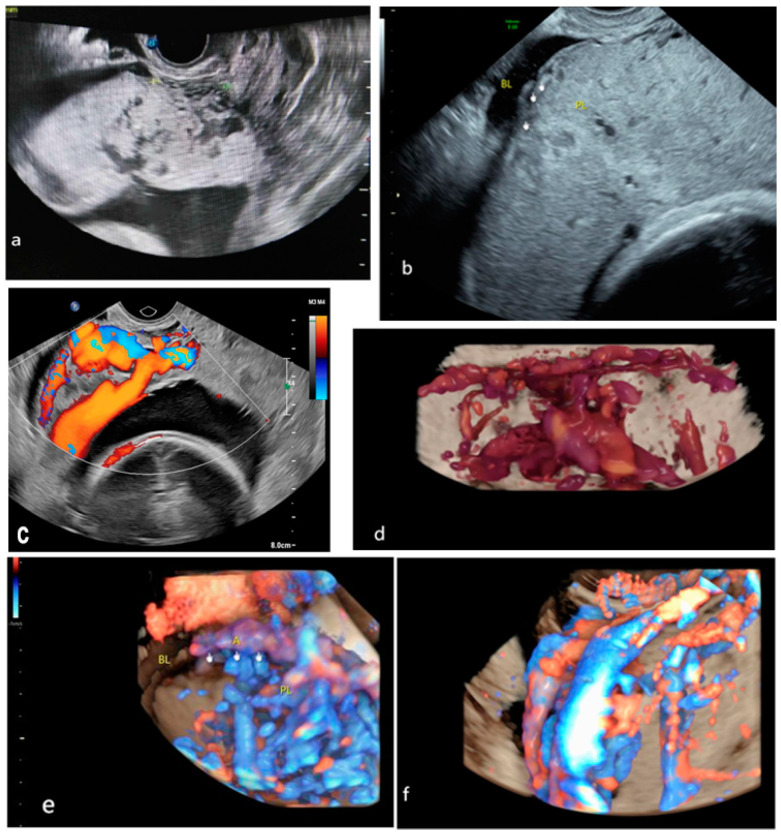
Common traditional ultrasound signs: (**a**) placental lacunae; (**b**) myometrial thinning; (**c**,**d**) bladder wall interruption; (**e**,**f**) bridging vessels. BL, bladder. A, artery. PL, placenta.

The concept of placental lacunae scoring was developed to quantify the number and characteristics of anechoic areas within the placenta, which can indicate potential invasion [17]. With advancements in imaging technology, color Doppler and 3D ultrasound have been increasingly incorporated into these scoring systems, thereby enhancing their sensitivity and detection capabilities. Modern ultrasound scoring systems evaluate the risk of PAS by quantifying several key features, including the following: placental lacunae, the hypoechoic zone behind the placenta, the myometrial thickness, the bladder wall line, local exophytic masses, the blood flow in the uterine–bladder region, rich blood flow below the placenta, and turbulent blood flow within the placenta (Figure 2; Table 1).

These scoring systems quantify multidimensional ultrasound signs, thereby helping to accurately predict the likelihood and degree of PAS invasion [10,20,21].

### 3.4. New Ultrasound Signs for Diagnosing Placenta Accreta Spectrum

In recent years, advancements in ultrasound technology have significantly enhanced the diagnosis of PAS. The introduction and application of novel ultrasound signs have further improved our ability to recognize invasive placentation. While traditional features such as placental lacunae and myometrial thinning provide important preliminary diagnostic information, new signs such as the “rail sign [22]” and “placental bulge” have markedly increased the ability of screening tools to detect deep and complex cases (Table 2). Moreover, the use of color Doppler to observe the abnormal distribution of blood flow and the integration of 3D imaging technology enable the anatomical relationship between the placenta, uterine wall, and neighboring organs to be interpreted more comprehensively [16].

**Table 1:** Definitions of traditional signs of placenta accreta spectrum on ultrasound.

Traditional Ultrasound Sign	Description
Placental Lacunae	Placental lacunae are irregular hypoechoic or anechoic areas within the placenta, often indicating invasive placental growth [23].
Myometrial thinning	Myometrial thickness < 1 mm or undetectable [24].
Bladder wall interruption	The partial or complete interruption, or the loss or irregularity, of the bladder wall or hyperechoic line between uterine serosa and bladder lumen [23].
Loss of hypoechoic zone	The loss or irregularity of a normal hypoechoic interface between the uterine wall and placental basal plate [17].
Placental bulge	‘Ballooning’ of the uterus containing the placenta into the surrounding pelvic structure [23].
Abnormal vascularity	The disruption of blood flow patterns in the placenta and the uterine wall [10].
Bridging vessels	Vessels appear to extend from the placental bed, across the uterine wall into the bladder or other pelvic organs [10,23,25].

### 3.5. Application of Intraoperative Ultrasound

During delivery, ultrasound allows doctors to monitor the relationship between the placenta and the uterine myometrium, serosa, and adjacent organs such as the bladder and rectum in real time. Color Doppler ultrasound facilitates the real-time observation of abnormal blood flow patterns, guiding hemostatic procedures and informing surgical decisions. Intraoperative ultrasound provides guidance for the surgical team, reducing the risk of massive bleeding and hysterectomy. In complex invasive cases, it helps locate the invasive areas and supports the implementation of conservative surgery, such as partial resection of the uterus.

In summary, ultrasound can aid in the diagnosis of PAS due to its convenience, high availability, real-time dynamic observation capabilities, and non-radiative nature, making it the primary choice for clinical screening. It enables the rapid assessment of placental location, morphology, and abnormal blood flow distribution, which is particularly valuable for early screening and routine antenatal checks. However, ultrasound diagnosis is highly dependent on the operator’s expertise, and the imaging quality may be influenced in cases that involve a posterior placenta, complex anatomical structures, or maternal factors. Moreover, ultrasound has a limited ability to assess the depth of placental invasion and the involvement of adjacent organs, and its sensitivity to early pathological changes and microstructures is relatively low. For complex cases or when ultrasound images are unclear, the performance of MRI and other advanced imaging techniques is often necessary to enhance diagnostic accuracy.

## 4. Application of MRI in Diagnosing Placenta Accreta Spectrum (PAS)

The use of MRI in diagnosing PAS has gained prominence due to its ability to provide detailed anatomical information, particularly in cases where ultrasound findings are inconclusive or limited by technical constraints. Ultrasound, while highly effective in early screening, can be hindered by factors such as maternal obesity, the posterior placental location, or excessive bowel gas, which may obscure critical imaging details [29,30,31,32,33]. In contrast, MRI offers superior soft tissue contrast and high-resolution imaging, making it an invaluable tool for assessing the depth of placental invasion, myometrial thinning, and the involvement of adjacent organs. Additionally, MRI is particularly useful in complex cases, such as placenta percreta or multiple gestations, where the anatomical relationships are more intricate and require precise preoperative planning. As a result, MRI has become an essential complementary imaging modality, especially in high-risk pregnancies where the optimization of maternal and fetal outcomes relies upon accurate diagnosis. The diagnostic utility of MRI varies across different stages of pregnancy, with distinct imaging features emerging as the placenta develops [3,33,34,35]. In early pregnancy (11–14 weeks), MRI is particularly valuable for identifying early signs of placental invasion, such as the loss of the placenta–myometrium boundary and abnormal vascular signals. As pregnancy progresses into the second and third trimesters, MRI’s ability to depict the depth and extent of placental invasion becomes even more pronounced, providing critical information for surgical planning and risk stratification. The following sections will explore the specific features of MRI and its diagnostic applications in each trimester, highlighting its role in enhancing the accuracy and comprehensiveness of PAS diagnosis.

### 4.1. Application of MRI in Early Pregnancy (11–14 Weeks)

During early pregnancy (11–14 Weeks), MRI leverages its high-resolution imaging capabilities and superior soft tissue contrast to provide a clear visualization of the relationship between the placenta and uterine myometrium [2]. This modality is particularly advantageous for posterior placenta accreta or complex cases in which ultrasound images are inconclusive [31]. MRI can reveal the following features of PAS:

Placenta–myometrium boundary: T2-weighted imaging can show the loss or blurring of the boundary between the placenta and myometrium.

Placental thickening and irregular shape: MRI aids in identifying focal thickening and morphological irregularities in the placenta.

Abnormal vascular signals: Enhanced scans can detect abnormal blood flow, such as vascular proliferation and dilation at the placenta’s base. Other common signs include placental lacunae (low or no signal areas), myometrial thinning or absence, and abnormal protrusions of the placental edge [2,3,31,32,33].

However, in early pregnancy, the placenta is still in the developmental stage. The differentiation between decidua and placental tissue has not yet fully occurred, and the anatomical boundary between the placenta and myometrium is dynamically changing. As a result, trophoblast invasion or decidual damage may not yet present clear imaging features. Given that the placenta is relatively homogeneous in its shape and signals during early pregnancy, MRI may struggle to differentiate normal attachment from mild invasion. Additionally, key PAS features such as angiogenesis and blood flow abnormalities are still in the early stages of development, and may not exhibit significantly abnormal signals on MRI [3,31,32,33]. Moreover, damage to the myometrium in cesarean scar areas may not yet be fully reflected in typical signal changes, making it difficult for MRI to identify the depth of invasion or the integrity of the scar in early cesarean scar pregnancy lesions. Despite these limitations, MRI’s high resolution and superior soft tissue contrast remain valuable for assessing complex cases, especially when ultrasound results are inconclusive or when posterior placenta accreta is suspected [2,30].

### 4.2. Application of MRI in the Second and Third Trimester

**Figure 3:**
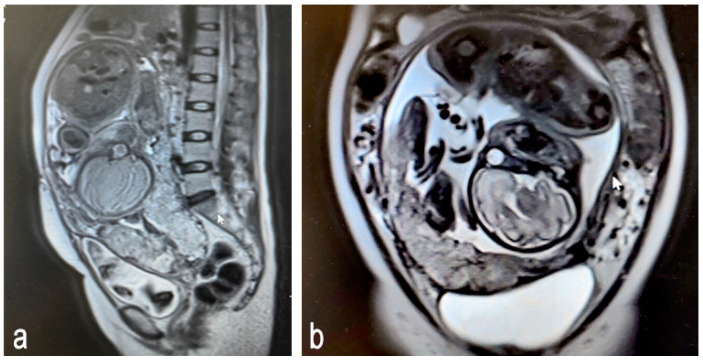
Magnetic resonance images of placenta accreta spectrum (Prenatal stage): (**a**) sagittal plane; (**b**) frontal plane.

In the second and third trimesters, the placenta has fully developed, allowing MRI’s high resolution to accurately depict the depth and extent of placental invasion. Compared to ultrasound, MRI provides clearer anatomical structural information, thus revealing detailed relationships [36].The common features of PAS detected using MRI include the loss or blurring of the placenta–myometrium boundary: in cases of abnormal attachment or invasive implantation, the normal boundary (low signal line) between the placenta and myometrium becomes blurred or disappears completely. T2-weighted images show the loss of definition between the placenta and myometrium. Severe cases may show direct penetration through the myometrium (Figure 3).

#### 4.2.1. Myometrial Thinning or Absence

Placental invasion results in significant thinning or the local absence of the myometrium, a critical diagnostic feature of PAS. The complete loss of the muscle layer indicates severe placenta percreta. On T2-weighted images, these invasive regions exhibit abnormal signal intensities and a marked reduction in thickness [31,37].

#### 4.2.2. Abnormal Blood Vessels at the Placental Base

PAS is frequently associated with vascular proliferation, dilation, and distribution abnormalities at the placental base. Enhanced MRI is able to detect these abnormal blood flow signals, which signify invasive angiogenesis and correlate closely with the depth of PAS invasion. Enhanced MRI shows abnormal high-signal vessels in the placental base, appearing as a network of proliferating blood vessels or dilated vessels [32,34,38].

#### 4.2.3. Placental Lacunae

Placental lacunae are an important imaging feature of PAS, appearing as irregular low-signal or no-signal areas within the placenta and indicating abnormal vascular expansion. On T2-weighted images, multiple irregular low-signal or no-signal areas of varying sizes and uneven distribution are visible within the placenta, resembling “Swiss cheese-like” changes [3,39].

#### 4.2.4. Abnormal Placental Edge

Invasive placentas often exhibit abnormal edges that protrude toward the serosa or penetrate adjacent organs, lacking the normal tapered transition and appearing curled or outwardly protruding [3].

#### 4.2.5. Uterine Shape Changes

Placental invasion can cause localized uterine bulging or morphological changes that are visible on MRI as bulging in the implanted area, with blurred boundaries or abnormal signals in nearby tissues [3,33].

#### 4.2.6. Abnormal Uterine–Bladder Interface

Severe PAS may involve placental penetration into the bladder or other adjacent organs, leading to the loss of distinct boundaries. Enhanced MRI shows abnormal signals at the uterine–bladder interface, bridging vessels, and irregular thickening or abnormal signals in the bladder wall [33,40].

#### 4.2.7. Placental Thickness and Signal Changes

Invasive placentation typically shows increased thickness and mixed signal changes on T2-weighted images, with localized thickening and areas of high and low signals [41].

#### 4.2.8. Placental Invasion of Other Organs

In placenta percreta, the placenta may invade organs such as the bladder or rectum, with enhanced MRI showing abnormal signals and tissue destruction in these areas [3,13].

However, MRI does not monitor blood flow dynamics in real time as effectively as color Doppler ultrasound and may be less sensitive for mild lesions, thus limiting its early diagnostic role in certain cases.

### 4.3. Recent Developments in MRI for Diagnosing Placenta Accreta Spectrum

#### 4.3.1. Application of High-Field MRI

Traditionally, 1.5T MRI systems have been used for uterine imaging and the diagnosis of PAS. 3.0T MRI offers a superior spatial resolution, providing clearer images of soft tissues. This is particularly beneficial when evaluating the intricate relationship between the placenta, uterine myometrium, and adjacent tissues such as the bladder and rectum. In the second and third trimesters, 3.0T MRI can more clearly depict signs such as placental thickening, vascular abnormalities, and the blurring of the placenta–myometrium boundary. These features are crucial for assessing placenta percreta, where the placenta invades the uterine muscle, serosal layer, or adjacent organs, and thus offer more precise imaging support [42].

#### 4.3.2. Introduction of Functional MRI Techniques

With the advancement of MRI technology, functional MRI has been widely used for the early diagnosis of PAS, particularly in evaluating the microstructure, blood flow, and tissue changes in the placenta.

#### 4.3.3. Diffusion-Weighted Imaging (DWI)

DWI uses water molecule diffusion to reflect microcirculation. In PAS, structural changes in invasive placental tissue restrict water diffusion. DWI detects abnormal microcirculation and differentiates normal placental tissue from invasive areas, which is crucial for identifying deep invasion, ischemic changes, or microvascular expansion [34,43].

#### 4.3.4. Intravoxel Incoherent Motion (IVIM)

IVIM is an emerging MRI technique that detects microvascular perfusion changes, distinguishing normal placental tissue from invasive regions. By monitoring blood perfusion, IVIM identifies abnormal blood flow and assesses early signs of placental invasion and angiogenesis [34].

#### 4.3.5. Diffusion Kurtosis Imaging (DKI)

DKI captures complex water molecule movements with higher sensitivity to microstructural abnormalities than conventional DWI. It excels in detecting microscopic changes in the placenta, aiding in the early recognition of invasive PAS and identifying deep tissue invasion [34].

#### 4.3.6. Integration with Artificial Intelligence (AI) and Automated Analysis

AI-assisted automated image analysis rapidly identifies key PAS features such as placental thickening, vascular proliferation, and lacunae, improving diagnostic efficiency and accuracy. AI enhances both the sensitivity and the specificity, providing standardized and quantitative image analysis that is especially beneficial in multicenter diagnoses [44].

### 4.4. Comparison of Ultrasound and MRI in Diagnosing PAS

Both ultrasound and MRI play complementary roles in diagnosing the placenta accreta spectrum (PAS), and each exhibits strengths and limitations (Table 3).

## 5. Other Imaging Techniques in Diagnosis

While ultrasound and MRI are the tools most commonly employed to diagnose PAS, other techniques such as CT and angiography can be used prenatally. Although rarely used for prenatal diagnosis, CT can clearly show the anatomical relationship between the placenta and surrounding organs, thus aiding in preoperative planning by evaluating the relationships between organs and the extent of invasion. CT angiography helps identify abnormal blood vessels and assess the risk of bleeding. However, due to its use of radiation, limited soft tissue resolution, and potential impact on the fetus, CT should be used with caution [13,32].

Angiography assesses the vascular relationships between the placenta, uterus, and adjacent organs, determining the extent of invasion and mapping abnormal blood vessels for surgical planning. However, due to its invasiveness and use of radiation, angiography should be utilized with caution.

## 6. Diagnostic Value and Future Directions

Ultrasound and MRI are key imaging techniques for the prenatal diagnosis of PAS.

Preferred initially due to its convenience, real-time observation, and lack of radiation, ultrasound is ideal for early screening and low-risk cases. Its accessibility, cost-effectiveness, and real-time evaluation of placental location, morphology, and blood flow are particularly advantageous. However, its use is limited in cases with posterior placenta accreta, in individuals with a complex anatomy, and in obese patients, and heavily depends on the operator’s skill. With technological advancements, ultrasound applications have expanded. Research now focuses on AI-assisted diagnosis, elastography, and contrast-enhanced ultrasound, enhancing the sensitivity of early detection. Portable devices and real-time 3D imaging will further extend its use, especially in community hospitals and remote areas.

Alongside ultrasound, MRI excels in assessing the depth of placental invasion, the relationships with surrounding organs, and microstructural features. It provides high-resolution soft tissue images, which are crucial for evaluating the invasion of the myometrial or serosal layer, particularly in cesarean scar pregnancies or complex cases. Functional MRI techniques such as DWI, DKI, and IVIM enhance assessments of the placental microstructure and blood flow. Future developments in super-high-field MRI and multimodal imaging will offer more comprehensive diagnostic solutions.

The FIGO classification divides PAS into three grades—placenta accreta, placenta increta, and placenta percreta—based on the degree of placental invasion into the myometrium, with each grade associated with an increasing risk of bleeding during surgery [45]. Ultrasound and MRI are crucial for assessing the severity of PAS. Placenta accreta is characterized by abnormal attachment without deep invasion, which can be detected through ultrasound by identifying myometrial thinning and increased blood flow. As the condition progresses to placenta increta and placenta percreta, the placenta invades deeper into the myometrium and beyond, exhibiting features such as placental lacunae and vascular abnormalities, which can be more clearly assessed with MRI. These imaging features, including increased vascularity and placental lacunae, correlate directly with the severity of PAS and the risk of significant hemorrhage. By combining ultrasound and MRI, clinicians can perform a comprehensive assessment of the extent of placental invasion, predict blood loss, and guide surgical planning to ensure timely and effective intervention. For instance, a multicenter study by Hessami et al. reported that early ultrasound screening in high-risk populations reduced the incidence of severe maternal complications by 30% [5]. MRI has been shown to improve surgical planning, with studies indicating that preoperative MRI assessment can reduce intraoperative blood loss by up to 50% in cases of placenta percreta and improve neonatal outcomes by reducing preterm birth rates by 20% [3,30].

The diagnostic performance of ultrasound (US) and magnetic resonance imaging (MRI) varies depending on the clinical context and the stage of pregnancy. When used individually, ultrasound demonstrates a sensitivity of 85–90% and a specificity of 78–82% for detecting PAS, particularly in early pregnancy [5,15]. MRI, on the other hand, offers superior soft tissue contrast and is particularly useful in complex cases, with a sensitivity of 89–92% and a specificity of 85–88% [3,30]. The combination of both modalities has been shown to further enhance diagnostic accuracy, with a sensitivity of 94% and a specificity of 90% [23].

However, this review primarily included studies from high-resource settings, which may limit its applicability to low-resource environments where access to advanced imaging technologies is limited. Additionally, the review did not address the potential cost-effectiveness of combining ultrasound and MRI, which is an important consideration for healthcare systems with limited resources. Future studies should explore the cost–benefit ratio of using both modalities in different healthcare settings.

## 7. Conclusions

The prenatal diagnosis of PAS focuses on the use of ultrasound and MRI. While ultrasound remains the preferred screening tool due to its non-invasive nature, convenience, and real-time diagnostic capabilities, its accuracy is influenced by operator skill and equipment quality. MRI serves as a valuable complementary tool, particularly in complex cases, providing detailed anatomical information and assessing the depth and extent of placental invasion. The combination of ultrasound and MRI offers a comprehensive diagnostic approach, significantly improving early detection and surgical planning, supporting effective management in high-risk pregnancies.

As technology advances, combining ultrasound and MRI will enable the precise diagnosis of PAS, improving the early detection and management of this high-risk condition.

## Figures and Tables

**Table 2:** Definitions of new ultrasound signs of placenta accreta spectrum.

New Ultrasound Sign	Description	Sensitivity	Specificity
Rail sign [22]	Sub-placental or uterovesical hypervascularity and neovascularization over the bladder mucosa usually involve vessels that are parallel and interconnected with perpendicular bridging vessels.	85%	82.3%
Pulsatile vessel at posterior bladder wall [26]	B-mode ultrasound shows the vessels as hypoechoic structures, and color Doppler imaging detects a pulsatile blood flow.	100%	/
Separation sign [27]	The separation sign indicates normal placental attachment when the placenta and uterine wall are clearly separated, with an intact hypoechoic zone, suggesting no placental invasion.	88.9%	100%
Intracervical lakes [28]	Intracervical lakes are defined as tortuous anechoic spaces within the cervix that appear to be hypervascular on color Doppler.	31%	95.0%
High acoustic radiation force impulse imaging elastography scores [19,23]	Shear-wave elastography velocity evaluation of placental stiffness (mean > 1.92 m/s).	58%	80%

**Table 3:** Comparison of ultrasound and MRI in diagnosing PAS.

Aspect	Ultrasound	Magnetic Resonance Imaging
Accessibility	Widely available in most healthcare settings, including low-resource areas.	Limited availability in resource-constrained settings; typically found in advanced or specialized centers [3,7].
Cost	Cost-effective and significantly less expensive than MRI.	Expensive, making it less feasible for routine screening or use in resource-limited settings [3,6].
Real-Time Imaging	Provides real-time dynamic assessment of blood flow and vascularity using color Doppler [24,26,38].	Cannot provide real-time blood flow assessment.
Diagnostic Speed	Fast and convenient; results can be obtained immediately.	Time-consuming, requiring longer scan durations [3].
Sensitivity in Early Pregnancy	High sensitivity for detecting early signs of PAS, such as placental lacunae, gestational sac location, and myometrial thinning [2,5].	Limited sensitivity in early pregnancy due to the homogeneous appearance of the placenta and dynamic anatomical changes [3,31,32].
Operator Dependency	Highly dependent on operator experience and technique, leading to variability in diagnostic accuracy [15].	Less operator-dependent; provides consistent imaging results across different radiologists if protocols are standardized.
Image Quality	Image quality may be affected by maternal obesity, excessive bowel gas, or posterior placenta location [29,30,31,32,33].	Not affected by maternal obesity or bowel gas; excels in posterior placenta assessment.
Visualization of Complex Cases	Limited in assessing depth of placental invasion or distinguishing between grades of PAS.	Superior in evaluating complex cases and identifying the depth of invasion, particularly for posterior placenta or multi-organ invasion [3,31,37].
Soft Tissue Contrast	Provides limited soft tissue contrast; subtle differences in tissue types may not be visible.	Offers excellent soft tissue contrast, allowing clear visualization of the placenta, myometrium, and adjacent organs [3,17,34].
Vascular Assessment	Allows dynamic vascular assessment using color Doppler; effective for identifying vascular proliferation and abnormal blood flow [24,26,38].	Enhanced MRI can detect vascular abnormalitiesbut lacks the real-time dynamic assessment capabilities of color Doppler ultrasound.
Safety	Non-invasive and safe for repeated use during pregnancy.	Gadolinium-based contrast agents may pose risks to the fetus and are generally avoided in pregnancy unless absolutely necessary [3].

## Data Availability

Data sharing is not applicable to this article as no new data were created or analyzed in this study.
